# Intake of Seven Essential Amino Acids Improves Cognitive Function and Psychological and Social Function in Middle-Aged and Older Adults: A Double-Blind, Randomized, Placebo-Controlled Trial

**DOI:** 10.3389/fnut.2020.586166

**Published:** 2020-11-25

**Authors:** Hiroyuki Suzuki, Daichi Yamashiro, Susumu Ogawa, Momoko Kobayashi, Daisuke Cho, Ai Iizuka, Masako Tsukamoto-Yasui, Michihiro Takada, Muneki Isokawa, Kenji Nagao, Yoshinori Fujiwara

**Affiliations:** ^1^Research Team for Social Participation and Community Health, Tokyo Metropolitan Institute of Gerontology, Tokyo, Japan; ^2^R&B Planning Department, Ajinomoto Co., Inc., Tokyo, Japan; ^3^Research Institute for Bioscience Products & Fine Chemicals, Ajinomoto Co., Inc., Kawasaki, Japan

**Keywords:** cognitive function, essential amino acids, psychosocial function, intervention study, a double-blind randomized placebo-controlled trial

## Abstract

**Background:** To delay the onset of dementia, it is important for healthy adults to take preventive actions before the cognitive function clearly declines. Protein malnutrition is a potential risk factor for senile dementia, although the precise link between protein/amino acid nutrition and cognitive function is unknown. The purpose of this study was to examine the effect of the ingestion of seven selected essential amino acids as a granular powder, namely, leucine, phenylalanine, and lysine supplemented with isoleucine, histidine, valine, and tryptophan on cognitive and psychosocial functions in healthy adults.

**Methods:** A double-blind, randomized, placebo-controlled trial was conducted. A total of 105 participants aged 55 years or older were randomly assigned to one of three groups: daily ingestion of 3 g (3gIG) or 6 g (6gIG) of the selected amino acids or daily ingestion of a placebo (PCG). Each group ingested the test powder for 12 weeks. As the main outcome, cognitive function was assessed before and after ingestion by a cognitive test battery. Psychosocial functions were also examined.

**Results:** The numbers of participants excluding dropouts were 35 in PCG and 3gIG and 33 in 6gIG. Analysis of covariance revealed that the 6gIG showed significantly improved cognitive function (Trail Making Test B), social interaction and psychological health scores after ingestion compared to the PCG (multiplicity adjusted *p* < 0.05).

**Conclusions:** Current findings suggested that ingestion of the seven essential amino acids led to improved attention and cognitive flexibility and psychosocial functioning, which is expected to prevent cognitive decline.

**Clinical Trial Registration:** University Hospital Medical Information Network Clinical Trial Registry (URL: https://upload.umin.ac.jp/cgi-open-bin/ctr/ctr_view.cgi?recptno=R000037779, Identifier: UMIN000033174).

## Introduction

The occurrence of dementia is increasing worldwide, and efforts promoting dementia prevention are important for building a society that can integrate people with dementia (1). There is currently no perfect pharmacological treatment to prevent dementia, so it is necessary to develop preventive methods that are accessible in daily life. Dementia is age related, associated with the accumulation of risk factors and protection factors over a lifetime. Even in healthy people before being diagnosed with dementia, cognitive decline can be a social problem. In order to delay the onset of dementia or reduce the cognitive decline, it is necessary to target middle-aged people and relatively young older people.

As a guideline for efforts to prevent dementia, Lancet's International Committee (1) and the World Health Organization (WHO) (2) have proposed lifestyle improvement measures. Improving nutrition is one of the lifestyle-based approaches that many people can adopt. Epidemiological studies on nutrition have shown the importance of protein intake for maintenance of brain function in older adults. A study on the relationship between caloric intake and cognitive decline has shown that high protein intake is associated with a reduced risk of mild cognitive impairment (MCI) or dementia (3). Recently, the relationship between self-reported dietary habits and biomarkers was also investigated in healthy older people, indicating that high protein intake has a potential protective effect against the amyloid beta burden in the brains of older adults (4).

Intervention studies have been conducted based on the viewpoint that protein intake maintains and improves cognitive function. In a 6-month intervention in which frail older adults consumed a protein drink, the study showed an improvement in reaction time measured by cognitive tests in the intervention group but no effect on other cognitive domains (5). To examine the effects of protein intake in detail, intervention studies focusing on amino acids have been carried out using supplements. A study in which prefrail older adults consumed supplementary L-carnitine, a type of amino acid, for 10 weeks showed an improvement in frailty index and grip strength but no improvement in cognitive function assessed by mini-mental state examination (MMSE) (6). It is considered that the intake of amino acids had a positive effect directly on the physical aspects. The change in cognitive function caused by the intervention may not have been measurable by a brief cognitive test such as MMSE. In a study using a neurocognitive battery to measure cognitive function, healthy older adults consumed supplements of the amino acid conjugates carnosine and anserine for 3 months, and the decline in verbal episodic memory measured by the Wechsler memory scale (WMS) was significantly lower in the intervention group than in the control group (7). Among protein treatments, the intake of amino acids seems to have a positive effect on cognitive function, but the intervention effect is not consistent.

The mechanism underlying the relationship between amino acid intake and neurocognitive function has been investigated in an intervention study in mice. In mice given a low-protein diet (LPD) for 2 months, impairment of learning and memory in cognitive function and agitation and disinhibition in behavior was observed (8). In relation to these abnormalities, there were deficiencies in neurotransmitters, such as gamma-aminobutyric acid, glutamate, glycine, dopamine, norepinephrine, serotonin, and aspartate, in the brains of LPD-fed mice. Oral administration of seven essential amino acids, namely, leucine, phenylalanine, and lysine, supplemented with isoleucine, histidine, valine, and tryptophan, hereinafter referred to as “Amino LP7” to LPD-fed mice improved cognitive function and behavioral symptoms. Intake of the seven essential amino acids confirmed that the amino acids complemented the neurotransmitter deficiency.

Similarly, in older humans whose protein intake decreases with age (9), intake of Amino LP7 may contribute to improvement in cognitive function. However, it is unclear in which cognitive region the effect is seen given the complexity of human cognitive function, unlike in mice. In addition, the improvements in agitation and disinhibition behaviors seen in mice are associated with psychological and social functions in humans. Since behavioral improvement is related to the subjective quality of life (QOL) of the person, this is an important intervention effect in older adults, in addition to the improvement in cognitive function.

Therefore, we conducted an intervention study in humans that required intake of Amino LP7, the efficacy of which was confirmed in a previous animal study. The participants in this study were middle-aged and older people who are the targets of dementia prevention. Human cognitive function consists of six areas: learning and memory, executive function, complex attention, language, perceptual-motor function, and social cognition (10). The evaluation index measures cognitive function across multiple domains and evaluates psychological and social functions involved in daily life.

## Methods

### Ethical Approval

All participants provided informed consent for inclusion before participating in the study. This study was conducted with the approval of the Institutional Review Board and Ethics Committee of the Tokyo Metropolitan Institute of Gerontology (H30-1-21) and Ajinomoto Co., Inc. The safety assessment for the test food (2018-002) was independently performed by a medical doctor who was not involved in the analysis performed in this study.

### Study Design and Participants

**Figure 1:**
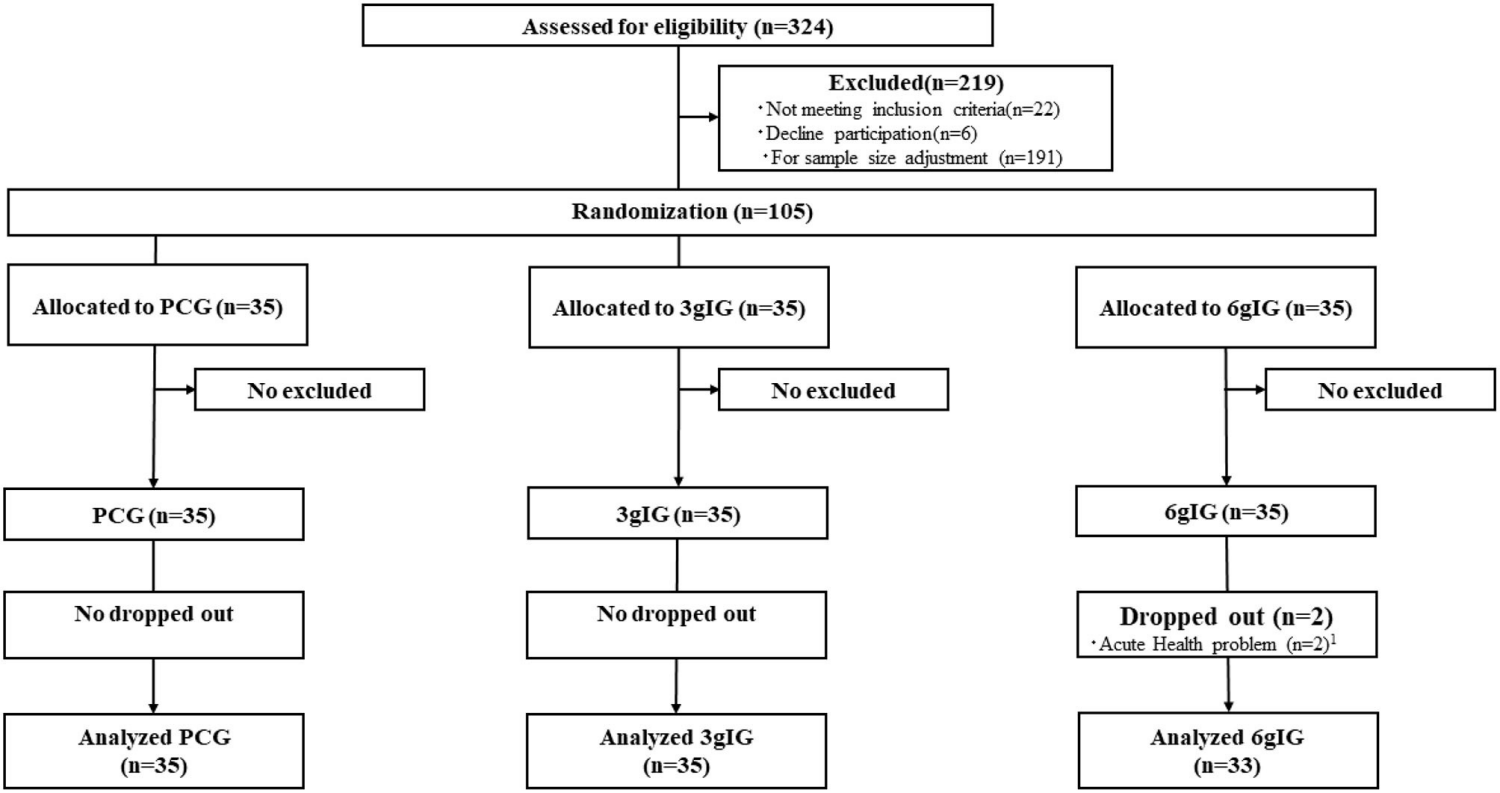
Consolidated Standards of Reporting Trials (CONSORT) flow diagram for the Amino LP7 intervention. ^1^Acute health problems were unrelated to this study. PCG, Placebo control group; 3gIG, Intervention group who takes amino acid 3 g per day; 6gIG, Intervention group who takes amino acid 6 g per day.

We conducted a double-blind, randomized, placebo-controlled trial in Tokyo. This study was registered in the University Hospital Medical Information Network Clinical Trial Registry (UMIN000033174, https://upload.umin.ac.jp/cgi-open-bin/ctr/ctr_view.cgi?recptno=R000037779). The Consolidated Standards of Reporting Trials (CONSORT) flow diagram (11) is presented in Figure 1.

Healthy participants in this study were recruited from individuals aged 55 years or older who had registered for a private clinical trial institution panel. For the initial recruitment, an invitation e-mail with the following conditions for participating in this study was sent to the individuals, and those who consented to the conditions were asked to participate in the screening test. The conditions were as follows: the participants (1) had awareness of forgetfulness or had been found to have forgetfulness by someone, (2) must have the same eating habits as usual during the intervention period, (3) must not take any medications or supplements that may affect cognitive function during the intervention period (treatments for other illnesses are not restricted), and (4) must keep an intake record of the tested composition throughout the study and submit it.

After the initial recruitment, 324 individuals participated in the screening test. In the screening test, brief cognitive assessments [MMSE-J (12, 13), the Japanese version of the Montreal Cognitive Assessment-Japanese (MoCA-J) (14, 15) and a questionnaire-based assessment of depressive symptoms using the Geriatric Depression Scale-Japanese (GDS-J) (16)] were conducted. The participants' medical history and the presence of allergies were also noted.

The inclusion criteria were as follows: the participants (1) were aged 55 or older, (2) obtained a score of 26 or higher scores on the MMSE-J and 29 or lower on the MoCA-J, and (3) had awareness of forgetfulness or had been found to have forgetfulness by someone. We excluded individuals if they (1) had a history of mental disorder, cerebrovascular disease, or alcohol dependence; (2) scored 6 or higher on the GDS-J; (3) had taken medications regularly that may have affected the central nervous system, except for sleeping pills; (4) had taken supplements regularly that may have affected their cognitive function; (5) had a visual or auditory impairment that interfered with their daily life; (6) were allergic to dairy products; (7) were undergoing treatment for cancer or liver cirrhosis; (8) were on dialysis; (9) had phenylketonuria; (10) had participated or would participate in other clinical studies; or (11) were judged to be inappropriate as participants by a doctor.

The sample size was calculated based on a previous study (17). In this previous study, the difference in the effective score was ~5 after a 2-week intervention. In our study, after a 3-month intervention, the mean effect size was estimated to 7.5 by the linear change related to the intake period. The standard deviation was estimated as 11 in our study based on the previous study results (standard errors were ~2 in the groups of 30 participants). The required sample size was 105 (35 per group) based on the mean effect size and standard deviation as described above at the two-sided 5% significance level to achieve 80% power.

As a result of the screening, 28 individuals were excluded. To match the precalculated sample size (*N* = 105), 191 participants who passed the screening test were excluded. Finally, 105 participants were randomly assigned to three groups.

### Interventions

A granular powder that contained seven essential amino acids, namely, 0.47 g of leucine, 0.42 g of phenylalanine, 0.33 g of lysine hydrochloride, 0.13 g of isoleucine, 0.08 g of histidine hydrochloride, 0.06 g of valine, and 0.01 g of tryptophan, was put in a sachet; this ratio (Amino LP7) was suggested to be effective in a previous animal study (8). Based on the previous study conducted on mice, the applicable daily intake of Amino LP7 for humans was calculated to be between 3 and 6 g. Based on this calculation, the first group was asked to take 1.5 g of Amino LP7 twice a day, for a total of 3 g of Amino LP7 per day (3 g intervention group, 3gIG). The daily Amino LP7 intake of the 3gIG was as follows: 0.93 g of leucine, 0.85 g of phenylalanine, 0.66 g of lysine hydrochloride, 0.26 g of isoleucine, 0.16 g of histidine hydrochloride, 0.12 g of valine, and 0.02 g of tryptophan. The second group ingested twice the amount consumed by the first group at the same time and was required to take a total of 6 g of Amino LP7 per day (6 g intervention group; 6gIG). The daily Amino LP7 intake of the 6gIG was as follows: 1.87 g of leucine, 1.70 g of phenylalanine, 1.33 g of lysine hydrochloride, 0.52 g of isoleucine, 0.32 g of histidine hydrochloride, 0.23 g of valine, and 0.04 g of tryptophan. The third group was asked to take a composition that did not contain any amino acids (placebo control group, PCG). The participants in the PCG ingested the placebo (containing cornstarch and lactose) twice a day. All participants were asked to record their daily intake. The duration of the intervention period was set at 12 weeks.

### Measures

Cognitive assessments and questionnaires on psychosocial and daily function were administered before composition intake (pre) and 12 weeks after composition intake (post). The primary outcomes included the assessments of cognitive function. The secondary outcomes included the evaluation of psychological and social functions and safety evaluations.

### Baseline Characteristics

All participants were interviewed to obtain data on their baseline characteristics, including age, gender, years of education, mental health using a Japanese version of the WHO-Five Well-Being Index [WHO-5-J (18)], and functional capacity using the Tokyo Metropolitan Institute of Gerontology Index of Competence (TMIG-IC) (19). The WHO-5-J is an index of psychological health developed by the WHO. This scale consists of five items on a six-point Likert scale, which ask about mental status in the last 2 weeks. The answers for each item are assigned 0–5 points, and the total value is used as the score (range: 0–25), with higher scores indicating better mental health. The TMIG-IC consists of 13 multidimensional items classified under three subscales of instrumental self-maintenance, intellectual activity and social role.

### Assessment of Cognitive Function: Cognitive Function Battery

The following tests were conducted to evaluate each cognitive domain: The Logical Memory (LM) I and II tests, Digit Span Test (DST) and Visual Memory Span Test (VMST), all of which are subscales from the Wechsler Memory Scale Revised [WMS-R (20, 21)]; the Rey Auditory-Verbal Learning Test [AVLT (22)]; the Trail Making Test (TMT) Part A and Part B (23); verbal fluency tests (24); and Similarities, which is a subtest of Wechsler Adult Intelligence Scale-III [WAIS-III (25)].

The LM I and II tests are story recall tests that assess immediate and delayed verbal memory. These tests directly reflect everyday memory. The LM I test is an assessment of immediate memory. In this test, the examiner told a story, and participants were asked to recall the contents of the story immediately. Then, the examiner told a second story, and participants recalled the story. The LM II test is an assessment of delayed memory; the participants were asked to recall the two stories 30 min after the stories were read. Scores were calculated by adding the number of elements of the story that were recalled. The maximum score for both immediate and delayed recall is 50 points. Higher scores indicate better memory function.

The DST was used to evaluate verbal memory function. The DST is the sum of forward (Digit Span Forward; DSF) and backward (Digit Span Backward; DSB) task subscales. In the DSF, the participant is required to memorize and repeat a sequence of numbers. In the DSB, the participants are required to memorize a sequence and repeat it in reverse. The score ranges of the DST, DSF, and DSB are from 0 to 24, 0 to 12, and 0 to 12, respectively.

The VMST was used to evaluate visual memory function. The VMST is the sum of forward (Visual Memory Span Forward; VMSF) and backward (Visual Memory Span Backward; VMSB) task subscales. In the VMSF, the examiner touches random square sequences shown on a test paper, and the participants are required to repeat this sequence. In the VMSB, the participants are required to memorize and repeat the sequence in reverse. The score ranges for the VMST, VMSF, and VMSB are from 0 to 26, 0 to 14, and 0 to 12, respectively.

The AVLT was used to evaluate memory and learning function. In this test, 15 nouns (list A) were read by the examiner, followed by free recall by the participants five times consecutively. After the fifth recall, the examiner read another list of 15 new words, followed by free recall by the participants. Then, the participants were asked to carry out free recall of list A (immediate recall). Twenty minutes later, the participants were asked to again carry out free recall of list A (delayed recall).

TMT Part A (TMT-A) and Part B (TMT-B) were conducted to evaluate attention and executive function. TMT-A especially evaluates processing speed of the attention, and TMT-B especially evaluates working memory. Working memory involved in the performance of TMT-B includes cognitive flexibility in executive function and divided attention and alternating attention (22). Both parts of the TMT consisted of 25 scattered circles drawn on the examination paper. In TMT-A, the circles were numbered from 1 to 25, and the participant was asked to draw lines to connect the numbers in order as quickly as possible. In TMT-B, the circles included either numbers from 1 to 13 or the first 12 letters of the Japanese Hiragana alphabet. Participants were required to connect the numbers and letters alternately. Faster performance in these examinations indicates higher attention and executive function. Perceptual-motor function is also involved in the TMT, as participants need to coordinate the processing of visual information with the rapid movement of their fingers.

Verbal fluency tests in which participants are asked to generate as many words as possible according to prescribed cues within a minute were used as verbal function measurements. Phonemic fluency was assessed using the letters “Ka” and “Ho,” and semantic fluency was assessed using the categories “Animals” and “Vegetables.” The total number of words appropriately generated was considered the score.

The Similarities subtest of WAIS-III was conducted to evaluate language comprehension and logical categorical thinking ability. In this task, the examiner verbally presents two words with a common concept, and participants are asked how these words are similar. The score range for this task is from 0 to 33.

### Evaluation of Psychological and Social Functions

Psychological and social functions were evaluated with WHO-5-J, SF-36 (26–28), the Japan Science and Technology Agency Index of Competence to Assess Functional Capacity [JST-IC (29)], and the abbreviated version of the Lubben social network scale [LSNS-6 (30)] and based on the anxiety of forgetfulness.

SF-36 was used to measure health-related QOL. SF-36 is composed of 36 questions and, when scored, yields 8 domains. Ten items assess limitations in physical activities (Physical functioning), 4 items assess problems with work or other daily activities as a result of physical health problems (Role physical), 2 items assess limitations due to pain (Bodily pain), 5 items assess personal health and the expectation of changes in health (General health), 4 items measure energy and tiredness (Vitality), 2 items examine the effect of physical and emotional health on normal social activities (Social functioning), 3 items measure problems with work or other daily activities as a result of emotional problems (Role emotional), and 5 items assess happiness, nervousness and depression (Mental health). All domains were scored from 0 to 100 based on the Japanese national standard score, and higher scores showed better QOL.

JST-IC was used to measure higher vital function. JST-IC consists of four categories regarding “Technology usage,” “Information practice,” “Life management,” and “Social engagement.” Each category has four questions that can be answered with “yes” or “no.” The score range for each category is from 0 to 4 points, with higher scores indicating higher competence.

LSNS-6 was used to assess social interaction and to screen for social isolation. Three of the LSNS-6 items relate to kinship ties, and the remaining three relate to non-kin ties. The total scale score is the sum of all items (range: 0–30).

In addition to the above scales, we asked participants one question regarding anxiety of forgetfulness (“Are you anxious about forgetting things now?”) that can be answered with “yes,” “a little,” or “no.” The answer was given 1–3 points, with a higher score indicating stronger anxiety.

### Safety Evaluation

Clinical laboratory values were measured before and after the intervention period to assess the safety of Amino LP7. The following blood parameters were measured: white blood cell (WBC) count, red blood cell (RBC) count, hemoglobin (Hb), hematocrit (Ht), platelet count, total protein, albumin, aspartate aminotransferase, alanine aminotransferase, lactate dehydrogenase, total bilirubin, alkaline phosphatase, γ-glutamyl transpeptidase, urea nitrogen, creatinine, uric acid, sodium, chlorine, potassium, total cholesterol, LDL cholesterol, HDL cholesterol, triglyceride, fasting plasma glucose concentration (FPG), and glycated hemoglobin (HbA1c). To determine if the baseline concentrations of blood amino acids and metabolites changed, these concentrations were measured under fasting after the intervention. The urine parameters (urine protein, urine glucose, and urine occult blood) were measured before and after the intervention. Systolic and diastolic blood pressure, pulse rate, and body mass index (BMI) measurements were conducted before and after intervention.

### Statistical Analysis

The full study population was analyzed based on the intention-to-treat concept. The main analysis was the analysis of covariance (ANCOVA) to compare the 3gIG and 6gIG to the PCG. The covariates were age, gender, education level, BMI, and diastolic blood pressure, which are expected to affect cognitive function, and the pre-value of each dependent variable. The education level was classified as “1” for high school and vocational college and “2” for junior college, university, and graduate school. In the subanalysis, one-way analysis of variance (1-way ANOVA) was used to compare the PCG, 3gIG, and 6gIG. Statistical tests for each endpoint were conducted at the two-sided significance level of 0.05. Multiple comparisons between groups were conducted using Dunnett's method. In this exploratory analysis, the effects of Amino LP7 were judged by both the estimates of mean differences and the statistical results because of the issue of statistical multiplicity between outcomes. These interpretations of the statistical results were consistent with the view of the American Statistical Association (31). In addition, we examined the probability that the obtained statistical results would appear in the situation where Amino LP7 was not effective and have discussed the interpretation of the results. All statistical analyses were performed using R (ver.3.6.3).

## Results

### Compliance With the Intervention

The mean intake rate of the compositions was 99.1%. Two participants in the 6gIG group dropped out for health reasons unrelated to this study.

### Characteristics of the Participants

Table 1 shows the characteristics of the PCG [*N* = 35, mean ± standard deviation (SD) age 64.5 ± 4.2), 3gIG (*N* = 35, mean ± SD age: 64.1 ± 5.0] and 6gIG (*N* = 33, mean ± SD age: 64.1 ± 5.9). There were 49 participants with a MoCA-J score of <26 and a potential MCI (17 in PCG, 18 in 3gIG, 14 in 6gIG). There were no significant differences observed in age, gender, education, MMSE-J, MoCA-J, WHO-5-J, and TMIG-IC among the three groups.

**Table 1:** Baseline characteristics of participants.

**Mean (SD)**	**PCG**		**3gIG**		**6gIG**	
	***n*** **=** **35**		***n*** **=** **35**		***n*** **=** **33**	
Age	64.5	(4.2)	64.1	(5.0)	64.1	(5.9)
Number of male (%)	18	(51.4)	18	(51.4)	16	(48.5)
Education	14.7	(2.2)	15.1	(1.8)	14.6	(2.4)
MMSE-J	29.2	(1.2)	29.2	(1.0)	29.2	(0.9)
MoCA-J	25.7	(1.6)	25.7	(1.5)	25.8	(1.6)
WHO-5-J	12.7	(3.0)	13.3	(3.9)	13.0	(4.0)
**TMIG-IC**						
Instrumental self-maintenance	5.0	(0.0)	5.0	(0.0)	5.0	(0.0)
Intellectual activity	3.5	(0.7)	3.7	(0.6)	3.8	(0.5)
Social role	3.4	(0.9)	3.3	(1.0)	3.7	(0.5)

*PCG, Placebo control group; 3gIG, Intervention group who takes amino acid 3 g per day; 6gIG, Intervention group who takes amino acid 6 g per day, MMSE-J, Japanese version of the Mini Mental State Examination, MoCA-J, Japanese version of the Montreal Cognitive Assessment-Japanese, WHO-5-J, Japanese version of the World Health Organization-Five Well-Being Index, TMIG IC, Tokyo Metropolitan Institute of Gerontology Index of Competence*.

### Analysis of the Effects of Intervention

**Figure 2:**
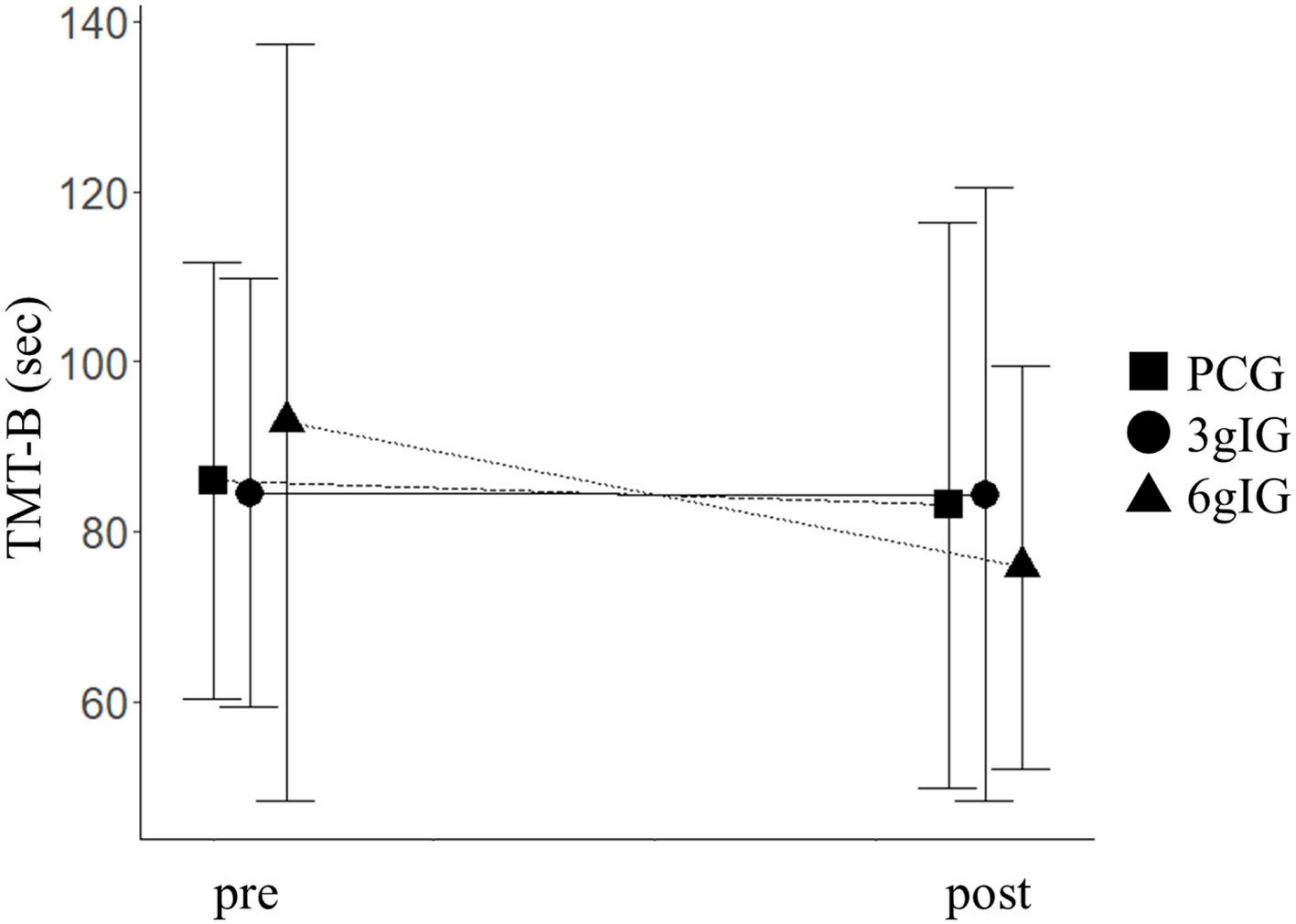
TMT-B before and after intervention for each group. PCG, Placebo control group; 3gIG, Intervention group who takes amino acid 3 g per day; 6gIG, Intervention group who takes amino acid 6 g per day. The mean values were plotted and the standard deviations were displayed as error bars.

As primary outcomes, scores of the dependent variables for cognitive function in each group are shown in Table 2. TMT-B showed a significant effect of the group based on ANOVA (*p* = 0.02). The time of TMT-B in the 6gIG group improved by 14.6 s compared to that in the PCG group (Figure 2). Based on the ANCOVA adjusted for multiplicity by Dunnett's method in our main analysis, the difference between PCG and 6gIG was significant (*p* = 0.04), while the difference between PCG and 3glG was not significant (*p* = 0.94). The results of other measurements of the cognitive function battery (WMS-R, AVLT, verbal fluency, and WAIS-III) were not significant between groups (LM I, LM II, DSF, DSB, DST, VMSF, VMSB, VMST, AVLT immediate recall, AVLT delayed recall, TMT-A, verbal fluency tasks, Similarities).

**Table 2:** The mean scores and ANCOVA results of cognitive tests before and after intervention.

		**Mean** ** (SD)**									**Residuals comparison (ANCOVA**^a^**)**					
											**Main effect of the group**		**Multiple comparisons by Dunnett's method**			
		**PCG**			**3gIG**			**6gIG**					**3gIG vs. PCG**		**6gIG vs. PCG**	
		**Pre**	**Post**	**Residual (post-pre)**	**Pre**	**Post**	**Residual (post-pre)**	**Pre**	**Post**	**Residual (post-pre)**	***F*-value**	***p*-value**	***t*-value**	**Adj. *p*-value**	***t*-value**	**Adj. *p*-value**
**WMS-R**																
Logical memory I (immediate)	Score (0–50)	22.71 (6.34)	26.74 (7.18)	4.03 (5.15)	23.89 (5.03)	27.00 (4.64)	3.11 (4.06)	23.70 (6.13)	27.70 (5.79)	4.00 (4.56)	0.68	0.51	−0.72	0.70	0.45	0.86
Logical memory II (delayed)	Score (0–50)	17.23 (6.89)	22.17 (7.2)	4.94 (4.17)	19.00 (6.75)	23.40 (6.01)	4.40 (4.63)	19.21 (6.50)	22.91 (7.38)	3.70 (5.92)	0.23	0.79	−0.07	1.00	−0.62	0.76
Digit span forward	Score (0–12)	8.14 (2.10)	7.91 (2.29)	−0.23 (1.65)	8.06 (2.04)	7.97 (2.13)	−0.09 (1.85)	8.48 (2.44)	7.88 (2.01)	−0.61 (1.89)	0.49	0.61	0.49	0.84	−0.52	0.83
Digit span backward	Score (0–12)	6.57 (1.96)	6.46 (1.98)	−0.11 (1.51)	6.77 (1.68)	6.49 (1.42)	−0.29 (1.53)	6.67 (1.69)	6.70 (1.70)	0.03 (1.51)	0.52	0.59	−0.31	0.93	0.70	0.71
Total of digit span	Score (0–24)	14.71 (3.82)	14.37 (3.75)	−0.34 (2.61)	14.83 (3.24)	14.46 (3.17)	−0.37 (2.73)	15.15 (3.70)	14.58 (3.27)	−0.58 (2.61)	0.01	0.99	0.15	0.98	0.03	1.00
Visual memory span forward	Score (0–14)	9.63 (1.88)	9.63 (1.66)	0.00 (1.59)	9.09 (1.52)	9.26 (1.77)	0.17 (1.84)	9.30 (1.67)	9.15 (1.77)	−0.15 (1.92)	0.26	0.77	−0.20	0.97	−0.71	0.70
Visual memory span backward	Score (0–12)	8.57 (2.03)	8.54 (1.77)	−0.03 (1.99)	7.94 (1.63)	8.23 (1.26)	0.29 (1.60)	8.12 (1.62)	7.91 (1.49)	−0.21 (1.92)	0.97	0.38	−0.40	0.89	−1.36	0.30
Total of visual memory span	Score (0–26)	18.20 (3.31)	18.17 (2.95)	−0.03 (2.93)	17.03 (2.61)	17.49 (2.39)	0.46 (2.50)	17.42 (2.78)	17.06 (2.62)	−0.36 (2.76)	0.85	0.43	−0.21	0.97	−1.22	0.37
**AVLT**																
Immediate recall	Score (0–15)	10.00 (3.43)	10.37 (3.15)	0.37 (3.38)	9.46 (2.33)	10.23 (2.87)	0.77 (2.82)	9.55 (3.15)	10.15 (2.53)	0.61 (2.36)	0.01	0.99	0.03	1.00	−0.10	0.99
Delayed recall	Score (0–15)	9.46 (3.51)	10.40 (3.42)	0.94 (3.18)	9.40 (2.57)	10.29 (1.93)	0.89 (2.25)	9.58 (2.94)	10.27 (2.76)	0.70 (2.35)	0.12	0.89	−0.33	0.92	−0.47	0.85
**Trail making test**																
Part A	Seconds to completion	41.80 (12.81)	38.17 (12.35)	−3.63 (10.70)	38.80 (13.48)	34.43 (13.5)	−4.37 (12.05)	39.42 (13.29)	36.03 (14.95)	−3.39 (14.27)	0.48	0.62	−0.98	0.52	−0.43	0.87
Part B	Seconds to completion	86.03 (25.72)	83.12 (33.24)	−2.44 (25.77)	84.51 (25.21)	84.35 (36.08)	−0.44 (23.49)	92.85 (44.58)	75.85 (23.72)	−17.00 (33.20)	4.05	0.02^*^	0.28	0.94	−2.34	0.04^*^
**Word fluency**																
Letter, “Ka”	Number of words	11.77 (4.45)	13.31 (4.23)	1.54 (3.68)	13.40 (2.96)	13.11 (3.66)	−0.29 (3.82)	13.24 (3.61)	14.55 (4.31)	1.30 (4.08)	2.52	0.09	−1.39	0.28	0.83	0.62
Letter, “Ho”	Number of words	9.09 (3.93)	9.43 (3.54)	0.34 (3.32)	9.34 (3.37)	9.51 (3.04)	0.17 (3.20)	9.88 (3.99)	10.33 (3.84)	0.45 (2.76)	0.46	0.63	−0.09	0.99	0.79	0.65
Animals	Number of words	17.69 (4.96)	18.11 (4.89)	0.43 (3.48)	18.63 (3.44)	17.46 (4.01)	−1.17 (3.54)	18.55 (3.83)	19.27 (5.09)	0.73 (4.31)	2.81	0.07	−1.57	0.21	0.78	0.66
Vegetables	Number of words	14.32 (4.23)	15.31 (4.41)	0.85 (2.58)	15.20 (3.39)	15.46 (3.90)	0.26 (3.70)	15.82 (4.42)	16.30 (4.46)	0.48 (2.81)	0.16	0.85	−0.48	0.84	0.02	1.00
**WAIS-III**																
Similarities	Score (0–33)	22.77 (4.49)	23.26 (4.70)	0.49 (2.58)	22.03 (4.60)	23.80 (3.26)	1.77 (3.68)	22.42 (4.06)	22.94 (3.72)	0.52 (3.29)	1.15	0.32	1.32	0.32	−0.01	1.00

a *Covariates: sex, age, blood pressure, BMI, education and value of pre-test. PCG, Placebo control group; 3gIG, Intervention group who takes amino acid 3 g per day; 6gIG, Intervention group who takes amino acid 6 g per day; WMS-R, Wechsler memory scale revised; WAIS-III, Wechsler Adult Intelligence Scale-III; ANCOVA, analysis of covariance; AVLT, auditory verbal learning test; BMI: body mass index*;

* *p < 0.05*.

As secondary outcomes, scores of the psychosocial function in each group are shown in Table 3. We conducted ANCOVA between groups for these dependent variables as well as the dependent variables for cognitive function. The results showed that WHO-5-J (*p* = 0.03), JST-IC Social engagement (*p* = 0.01), and LSNS-6 (*p* = 0.03) were significant. The ANCOVA adjusted for multiplicity by Dunnett's method in the main analysis showed that the differences between the PCG and the 3gIG were not significant for any of the above variables. On the other hand, the differences between the PCG and the 6gIG were significant [WHO-5-J (*p* = 0.03); JST-IC Social engagement (*p* = 0.01); LSNS-6 (*p* = 0.02)]. For other measured variables, the effects of the group were not significant by ANCOVA (SF-36 Physical functioning, SF-36 Role physical, SF-36 Bodily pain, SF-36 General health, SF-36 Vitality, SF-36 Social functioning, SF-36 Role emotional, SF-36 Mental health, JST-IC Technology usage, JST-IC Information Practice, JST-IC Life management, anxiety of forgetfulness).

**Table 3:** The mean scores and ANCOVA results of psychosocial functions before and after intervention.

		**Mean** ** (SD)**									**Residuals comparison (ANCOVA, covariance: sex, age, blood pressure, BMI, education, value of pre-test)**					
											**Main effect of** ** the group**		**Multiple comparisons by Dunnett's method**			
		**PCG (*****n*** **=** **35)**			**3gIG (*****n*** **=** **35)**			**6gIG (*****n*** **=** **33)**					**3gIG vs. PCG**		**6gIG vs. PCG**	
		**Pre**	**Post**	**Residual** ** (post-pre)**	**Pre**	**Post**	**Residual** ** (post-pre)**	**Pre**	**Post**	**Residual (post-pre)**	***F*-value**	**p-value**	***t*-value**	**Adj. *p*-value**	***t*-value**	**Adj. *p*-value**
**WHO five well-being index**	Score (0–25)	16.94 (3.21)	16.60 (3.38)	−0.34 (4.13)	16.71 (3.85)	16.89 (3.48)	0.17 (2.84)	16.73 (4.26)	18.30 (3.51)	1.58 (3.69)	3.49	0.03^*^	0.49	0.84	2.51	0.03^*^
**SF-36**																
Physical functioning	Score (0–100)	93.71 (7.21)	92.71 (6.79)	−1.00 (6.62)	89.00 (11.03)	92.57 (9.10)	3.57 (6.92)	88.64 (13.65)	91.21 (10.16)	2.58 (9.36)	1.74	0.18	1.86	0.12	0.88	0.58
Role physical	Score (0–100)	93.75 (9.94)	90.89 (12.06)	−2.86 (11.28)	91.07 (12.43)	91.61 (14.46)	0.54 (17.37)	91.29 (13.62)	92.42 (12.67)	1.14 (11.64)	0.42	0.66	0.63	0.75	0.88	0.58
Bodily pain	Score (0–100)	76.43 (16.77)	71.11 (15.53)	−5.31 (19.83)	78.31 (19.48)	73.69 (20.00)	−4.63 (21.42)	74.76 (17.57)	76.45 (16.77)	1.70 (13.11)	1.32	0.27	0.36	0.91	1.56	0.21
General health	Score (0–100)	73.94 (12.74)	73.09 (12.46)	−0.86 (13.71)	71.66 (14.19)	73.03 (16.06)	1.37 (9.32)	71.27 (13.21)	72.42 (13.20)	1.15 (8.57)	0.23	0.80	0.64	0.75	0.49	0.84
Vitality	Score (0–100)	70.54 (14.74)	69.64 (16.12)	−0.89 (17.49)	65.71 (12.63)	69.29 (13.59)	3.57 (13.24)	65.91 (13.54)	72.16 (13.98)	6.25 (11.9)	1.01	0.37	0.78	0.65	1.42	0.27
Social functioning	Score (0–100)	93.57 (11.89)	92.14 (14.57)	−1.43 (15.98)	93.21 (12.26)	91.43 (14.78)	−1.79 (14.27)	93.18 (13.29)	95.83 (8.65)	2.65 (12.79)	1.18	0.31	−0.07	1.00	1.30	0.33
Role emotional	Score (0–100)	92.14 (14.14)	92.38 (11.50)	0.24 (14.22)	92.62 (12.75)	93.10 (13.48)	0.48 (12.93)	91.67 (13.66)	92.68 (13.94)	1.01 (14.55)	0.11	0.90	0.39	0.90	0.40	0.89
Mental health	Score (0–100)	79.71 (11.18)	79.14 (10.95)	−0.57 (10.76)	79.29 (11.83)	78.57 (10.61)	−0.71 (10.99)	81.52 (11.62)	81.52 (10.72)	0.00 (9.6)	0.36	0.70	0.17	0.98	0.81	0.64
**JST-IC**																
Technology usage	Score (0–4)	3.86 (0.36)	3.97 (0.17)	0.11 (0.32)	3.91 (0.37)	3.86 (0.60)	−0.06 (0.24)	3.88 (0.33)	3.94 (0.24)	0.06 (0.24)	2.89	0.06	−2.38	0.04^*^	−0.89	0.58
Information practice	Score (0–4)	3.49 (0.85)	3.49 (0.70)	0.00 (0.8)	3.54 (0.74)	3.74 (0.51)	0.20 (0.76)	3.52 (0.80)	3.52 (0.67)	0.00 (0.61)	2.02	0.14	1.79	0.14	0.06	1.00
Life management	Score (0–4)	3.43 (0.70)	3.54 (0.74)	0.11 (0.63)	3.40 (0.77)	3.26 (0.89)	−0.14 (0.69)	3.48 (0.83)	3.58 (0.71)	0.09 (0.84)	2.28	0.11	−1.78	0.14	0.16	0.98
Social engagement	Score (0–4)	1.71 (1.43)	1.66 (1.35)	−0.06 (0.91)	1.74 (1.44)	1.77 (1.48)	0.03 (1.10)	1.67 (1.31)	2.30 (1.45)	0.64 (1.22)	4.42	0.01^*^	0.34	0.92	2.75	0.01^*^
**LSNS-6**	Score (0–30)	15.09 (6.22)	13.71 (5.77)	−1.37 (3.99)	14.03 (6.56)	14.20 (6.48)	0.17 (4.31)	15.33 (5.79)	16.06 (5.60)	0.73 (4.21)	3.50	0.03^*^	1.14	0.42	2.64	0.02^*^
**Anxiety of forgetfulness**	Score (1–3)	2.00 (0.54)	1.86 (0.55)	−0.14 (0.60)	2.06 (0.48)	2.03 (0.62)	−0.03 (0.57)	2.21 (0.60)	2.12 (0.60)	−0.09 (0.58)	1.03	0.36	1.26	0.35	1.21	0.38

*PCG, Placebo control group; 3gIG, Intervention group who takes amino acid 3 g per day; 6gIG, Intervention group who takes amino acid 6 g per day; JST-IC, The Japan Science and Technology Agency Index of Competence to Assess Functional Capacity; LSNS-6, The abbreviated version of the Lubben social network scale, ANCOVA, Analysis of covariance; BMI, body mass index*;

* *p < 0.05*.

By comparing the 6gIG and PCG, 4 out of 32 outcomes, including primary and secondary outcomes, showed significant results. In addition, these 4 outcomes showed consistently that the 6gIG showed a stronger effect than the PCG.

### Safety Evaluations

There were no abnormalities or fluctuations in clinical laboratory values, urine laboratory test results, vital data or BMI that could lead to safety concerns. The baseline concentrations of the seven essential amino acids included in this study did not increase under fasting. The assessment of safety was made through medical interviews with each subject, and no issues related to these interventions were reported. The participants who dropped out from the 6gIG did not do so because of side effects of the intervention.

## Discussion

The purpose of the present study was to examine the effect of intake of Amino LP7 on cognitive function as the primary outcome and psychosocial function as the secondary outcome in middle-aged and older adults. The results demonstrated that attention and cognitive flexibility assessed by TMT-B was improved in the 6gIG, whereas no significant changes were observed in either the 3gIG or PCG. An effect of intervention on TMT-B was observed, and the 15-s improvement of 6gIG was similar to the intervention effects reported by previous intervention studies (32, 33). The faster performance of the TMT-B suggests the improvement of the ability to concentrate on the task, pay attention to multiple tasks and memorize information needed for doing tasks, which is related with working memory. These results indicate that daily intake of 6 g of amino LP7 contributes to improved attention and executive function. In addition, psychological health and social interaction were also improved in the 6gIG group. A good WHO-5-J score indicates positive emotion (cheerful, active, fresh, interesting and vigorous emotion), and expanding social networks indicate improved social interaction. Although there was a potential limitation in the interpretation of the results because of multiple testing due to the study design, all 4 statistically significant results showed that Amino LP7 had positive effects only in the 6gIG. These results suggested that 6gIG may have an effect on cognitive function.

Two models can be considered for understanding why 6gIG was effective in improving attention and executive function and psychosocial function. The first model is that the intake of essential amino acids directly affects brain function through the transfer of amino acids to the brain. The effect of the intervention on attention and executive function in the 6gIG suggests that the intake of Amino LP7 affected frontal lobe function. If the intake of Amino LP7 had an effect on improving general health, there should have been an improvement in processing speed, as seen in an exercise-based intervention (34). Given that there was no change in processing speed and that the intervention had an effect on only working memory, intake of Amino LP7 perhaps had a specific positive effect on frontal lobe function. The second model is that Amino LP7 brings about improvement in psychosocial function, and changes in behavior in daily life led to improvement in attention and executive function. A study on social interactions showed that older people with more satisfactory interactions are at lower risk of developing dementia (35). An intervention study focusing on social interactions showed improvements in verbal memory and working memory due to the acquisition of new intellectual skills that accompany social interaction (36). In a study that examined the effects of social interaction among cognitive interventions, intervention via acquisition of new intellectual skills through analog games was found to affect visual working memory, and the effect increased with social interaction (37). It is also possible that intake of Amino LP7 first improved psychological health, which in turn led to improved social interaction and attention and executive function. Furthermore, it is possible that the two models mentioned above had a synergistic effect. No intervention effect was observed with 3gIG in either model, suggesting that additional doses of Amino LP7 were ineffective at low doses and that a dose of 6 g or more was required per day.

It is not possible to elucidate the mechanism based solely on the results of this study, which aimed to investigate the intervention effect of essential amino acid intake. Our initial hypothesis regarding this composition, Amino LP7 (8), was based on the amino acid influx rate to the brain, which would reflect the requirement for each amino acid to maintain brain homeostasis against neurodegenerative processes; we composed a mixture of seven essential amino acids rich in leucine, phenylalanine, and lysine to directly match the ratios associated with the brain influx rate. It is possible that Amino LP7 acts via multiple mechanisms, including neurotransmitter compensation (8) and competitive inhibition of neurotoxic substance influx into the brain. As the constituents of Amino LP7 have high rates of influx into the brain via specific transporters (e.g., LAT-1), it is plausible that Amino LP7 not only delivers the neurotransmitter substrate into the brain but also competitively inhibits the blood-to-brain transfer of toxic amino acid metabolites, such as kynurenine, that share the same transporters (38, 39). These metabolites are known to exert proinflammatory effects that induce neuroinflammation (40, 41), and thus, competitive inhibition of these neurotoxic metabolites may be involved in the efficacy observed herein. To clarify the mechanism underlying the improvement in attention and executive function via ingestion of Amino LP7, it is necessary to measure neurotransmitters and brain inflammation in human studies. As mentioned in the previous paragraph, the improvement of attention and executive function may be preceded by the improvement of psychosocial function. Of the seven amino acids used in this study, some amino acids such as tryptophan and phenylalanine may have antidepressant-like effects associated with mental health (42, 43). A study of community dwelling older adults in Japan found an association between frequency of going out and depression (44), and it is possible that prevention of depressive mood caused by tryptophan intake activated daily activities. In our study, the blood sample was collected under fasting condition to evaluate the safety of the intervention. Evaluating the dynamics of amino acid concentrations immediately after ingestion will help elucidate the mechanism in the future.

Based on the cognitive reserve hypothesis, it is possible to delay the onset of dementia by improving cognitive function in normal conditions (1, 45). Improvement of attention and executive function associated with frontal lobe function by ingestion of Amino LP7 may counteract not only the development of dementia due to Alzheimer's disease but also cognitive decline associated with aging. It would be of great significance if actual behaviors could be transformed to positive ones for health via easily accessible interventions such as supplements.

In the present study, it was possible to confirm the effect of intervention on attention and executive function, but there was no effect on learning function, which was observed in a previous study conducted in mice. The reason for this lack of effect was that the participants in this study were healthy adults with no impairment in learning function. To examine the effect of essential amino acid intake on learning function, it may be necessary to perform the experiment in terms of recovery of cognitive decline, not prevention of cognitive disorder. This finding may also be related to the lack of a deliberately created low-protein state. In human studies, when targeting cognitively impaired people or older persons with low-protein status, there is a possibility that intervention effects may be seen in learning, memory function and “behavioral and psychological symptoms of dementia (BPSD).”

No improvement in QOL or elimination of forgetfulness was observed in any of the intervention groups. It is suggested that short-term intervention with Amino LP7 for 3 months does not affect general subjective health in daily life. In the 6gIG, which showed an intervention effect on mental function and social interaction, subjective QOL may be improved by continuing the intervention.

This study has a few limitations. The first is that the improvement of social interaction was indicated by a questionnaire, and it was not possible to evaluate what kind of interaction was specifically affected. Although it is difficult to objectively evaluate social interaction, it is important to evaluate whether the effects of essential amino acid intake also affect the total amount of communication and daily enjoyment in order to enhance the significance of the intervention. The second point is that the intervention period was only 3 months long, and the long-term effects were not considered. From the perspective of dementia prevention, it is necessary to examine whether the effect of an intervention on attention and cognitive flexibility continues for a long duration. Moreover, it is not clear whether the intervention effect remains after intake is stopped. Long-term observational studies are also valuable in examining whether the efficacy of amino acids impacts the prevention of future cognitive decline. Third, due to the exploratory nature of this study, there should be a potential limitation in the interpretation of the results because of multiple testing due to the study design. However, if Amino LP7 was not effective, it is believed that both 6gIG and PCG would have shown effectiveness randomly. In the case where Amino LP7 was not effective, the probability that 4 out of 32 outcomes would show significant differences and that all 4 outcomes would be effective in only the 6gIG was only 0.8%. Our research results demonstrated that all 4 statistically significant results showed that Amino LP7 had positive effects in only the 6gIG group, suggesting the effectiveness of Amino LP7 on cognitive function. Further research with narrowed evaluation outcomes or larger clinical trials to adjust for the multiplicity of the outcomes is needed in the future.

In conclusion, daily intake of seven essential amino acids resulted in improved attention and cognitive flexibility and psychosocial functioning, but the effect required 6 g of daily intake. Intake of essential amino acids is associated with prevention of low protein status, although the mechanism underlying the intervention effect and the long-term effect are unclear. For older adults who need to work on not only cognitive decline but also frailty, health promotion through easily accessible methods such as supplements may be useful as an adjunct approach.

## Data Availability Statement

The datasets presented in this article are not readily available due to ethical and commercial restrictions but are available from the corresponding author on reasonable request.

## Ethics Statement

The studies involving human participants were reviewed and approved by the Institutional Review Board and Ethics Committee of the Tokyo Metropolitan Institute of Gerontology (H30-1-21) and Ajinomoto Co., Inc. The safety assessment for the test food (2018-002). The patients/participants provided their written informed consent to participate in this study.

## Author Contributions

MT-Y, MT, and HS: conceptualization, methodology, project administration, and writing—review and editing. KN: writing—review and editing. SO, DY, DC, and HS: data curation. SO, MI, MT-Y, and DY: formal analysis. SO, MK, DC, AI, MI, and HS: investigation. MT-Y and MT: resources. DY and MT: software. YF: supervision. SO, DY, and HS: visualization. MK, DY, and HS: writing—original draft. All authors have read and approved the published version of the manuscript.

## Conflict of Interest

MT-Y, MT, MI, and KN are employees of Ajinomoto Co., Inc., and HS received grants from Ajinomoto Co., Inc. The Amino LP7 and placebo were provided by Ajinomoto Co. Inc. The remaining authors declare that the research was conducted in the absence of any commercial or financial relationships that could be construed as a potential conflict of interest.
